# Comparison of complications and shocks in paediatric and young transvenous and subcutaneous implantable cardioverter-defibrillator patients

**DOI:** 10.1007/s12471-018-1186-1

**Published:** 2018-10-30

**Authors:** A. B. E. Quast, T. F. Brouwer, K. M. Kooiman, P. F. H. M van Dessel, N. A. Blom, A. A. M. Wilde, R. E. Knops

**Affiliations:** 1 Amsterdam UMC, Heart Centre, Department of Clinical and Experimental Cardiology, Amsterdam Cardiovascular Sciences, University of Amsterdam, Amsterdam, The Netherlands; 2 Amsterdam UMC, Department of Paediatric Cardiology, University of Amsterdam, Amsterdam, The Netherlands

**Keywords:** Implantable cardioverter-defibrillator, Device-related complications, Inappropriate therapy, Paediatric, Young, Subcutaneous ICD

## Abstract

**Background:**

Young implantable cardioverter-defibrillator (ICD) patients are prone to complications and inappropriate shocks (IAS). The subcutaneous ICD (S-ICD) may avoid lead-related complications. This study aims to describe the incidence and nature of device-related complications in young transvenous ICD (TV-ICD) and S‑ICD patients.

**Methods:**

Single-chamber TV-ICD and S‑ICD patients up to and including the age of 25 years implanted between 2002 and 2015 were retrospectively analysed. Complications were defined as device-related complications requiring surgical intervention. IAS were defined as shocks for anything other than ventricular tachycardia or ventricular fibrillation. Follow-up data were collected 5 years post-implantation. Kaplan-Meier estimates for complications at 5‑year follow-up were calculated with a corresponding 95% confidence interval.

**Results:**

Eighty-one patients (46 TV-ICD, 35 S-ICD) were included (median age 19.0 (IQR 16.0–23.0) and 16.5 (IQR 13.0–20.2) years respectively). Median follow-up was 60 and 40 months respectively. All-cause complication rate was 34% in the TV-ICD group and 25% in the S‑ICD group (*p* = 0.64). TV-ICD patients had more lead complications: 23% (10–36%) versus 0% (*p* = 0.02). The rate of infections did not differ between TV-ICD and S‑ICD: 2% (0–6%) versus 10% (0–21%) (*p* = 0.15). No systemic infections occurred in the S‑ICD patients. The rates of IAS were similar, TV-ICD 22% (9–35%) versus S‑ICD 14% (0–30%) (*p* = 0.40), as were those for appropriate shocks: 25% (11–39%) versus 27% (6–48%) (*p* = 0.92).

**Conclusion:**

The rates of all-cause complications in this cohort were equal, though the nature of the complications differed. S‑ICD patients did not suffer lead failures or systemic infections. An era effect is present between the two groups.

**Electronic supplementary material:**

The online version of this article (10.1007/s12471-018-1186-1) contains supplementary material, which is available to authorized users.

## What’s new?

- We evaluated device-related complications and inappropriate shock (IAS) therapy in paediatric and young subcutaneous (S-ICD) and transvenous (TV-ICD) implantable cardioverter-defibrillator patients. Due to the relatively recent introduction of the S‑ICD in 2009 a historical gap is present, i. e. not all patients had both devices available to them.
- Similar rates of device-related complications were seen in this young and paediatric patient cohort; however, the nature of device-related complications differed.
- We evaluated the incidence of IAS therapy. This cohort does not show a higher IAS rate for young S‑ICD patients than for TV-ICD patients.
- These data provide important insights for practising physicians to assist in their choice of device for this specific population. We found patients without an indication for pacing and a body weight above 30 kg to be most suitable for S‑ICD therapy, as similar complication rates occurred to those with TV-ICD therapy, with less invasive therapy, without risking systemic infections, and preserving venous access.

## Introduction

Implantable cardioverter-defibrillator (ICD) therapy in patients at high risk of sudden cardiac death (SCD) has proven to be effective [1]. In the paediatric and young adult population higher incidences of complications and inappropriate shocks (IAS) have been described, up to 22% and 20% respectively, compared with elderly patients [2, 3]. The number of children and young adults implanted with an ICD for primary prevention of SCD has increased due to improved genetic diagnoses for inherited arrhythmogenic diseases [4].

Transvenous (TV) ICD systems using intravascular leads may result in complications such as endocarditis, lead fractures or dysfunction, cardiac tamponade, perforation, pneumothorax and venous obstruction [5, 6]. A recent meta-analysis described lead malfunction as the most common device-related complication in young patients with inherited arrhythmia syndromes, showing a 10.3% lead malfunction rate during 4.5 years’ follow-up (2.3 annual rate) [2]. Lead failure results in the risk of undersensing a ventricular arrhythmia with the potential for arrhythmic death, as well as causing IAS in up to 60% of patients [5, 7]. Failed TV-ICD leads often, and infected leads always, require extraction with a risk of severe complications [8].

The subcutaneous ICD system (S-ICD) was developed to eliminate lead-related complications, but can only be used in patients who do not require pacing [9]. In the adult population, several studies have described a similar rate of IAS and device-related complications as with TV-ICDs, but fewer lead complications [7, 9, 10].

The objective of this study is to compare device-related complications, as well as appropriate and inappropriate therapy, in paediatric and young adult patients implanted with either TV-ICD or S‑ICD.

## Methods

### Study design and population

**Fig. 1:**
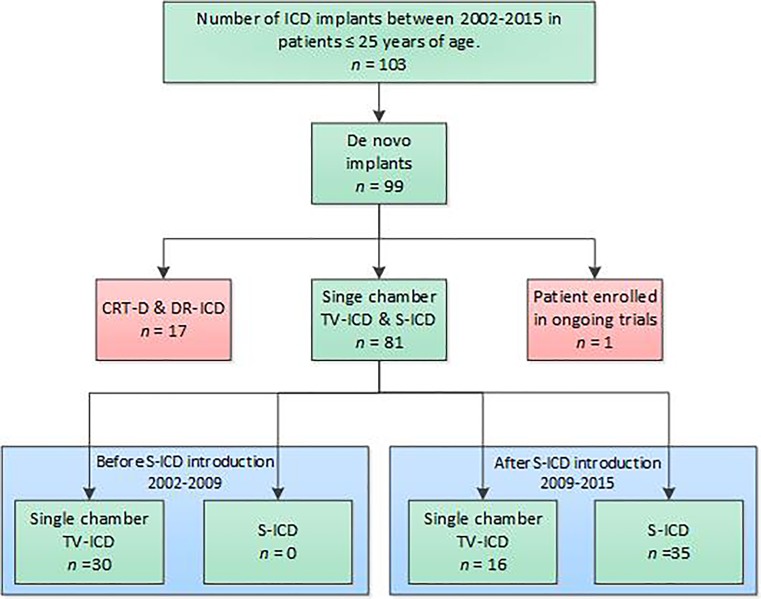
Patient selection flowchart (*ICD* implantable cardioverter defibrillator, *CRT-D* cardiac resynchronisation therapy—defibrillator, *DR—ICD* dual chamber ICD, *TV-ICD* transvenous ICD, *S-ICD* subcutaneous ICD, *VR-ICD* single chamber ICD; *PRAETORIAN, randomised trial comparing TV-ICD and S‑ICD in real life population [18])

This is a single-centre, retrospective study. The Institutional Review Board waived the need for informed consent. De novo single-chamber TV-ICD patients and S‑ICD patients up to and including 25 years of age, implanted in our centre between 2002 and 2015, were included (Fig. 1). Data were collected retrospectively: demographic details of the patients, diagnosis, ICD indication, chest radiographs and complications up to 5 years post-implantation. Implantation reports were used to collect data on conversion testing, ICD programming and implantation technique. After October 2010 all S‑ICD patients were implanted with the two-incision technique.

### Definition of endpoints

Device-related complications were defined as complications requiring surgical intervention. When available the electrogram was adjudicated by the investigators. Shocks were considered inappropriate when given for anything other than sustained ventricular tachycardia above the programmed lower rate zone or ventricular fibrillation.

### Statistical analysis

Continuous variables are expressed as median and interquartile range (IQR) or mean and standard deviation when normally distributed. Categorical variables are expressed as frequency with corresponding percentages. Continuous values were compared using the Mann-Whitney U test or unpaired *t*-test and categorical variables with Fisher’s Exact test. Kaplan-Meier (KM) rates for complications and appropriate therapy and IAS at 5‑year follow-up were compared with the log-rank test. A propensity score was calculated for each patient using logistic regression with device type as dependent variable and age at implant, height and weight as independent variables. Additionally Cox regression analysis was performed for device-related complications and ICD therapy correcting for propensity score. All reported *p*-values were 2‑tailed, and *p*-values <0.05 were considered statistically significant. Statistical analyses were performed in IBM SPSS Statistics 23 and R version 3.3.1.

## Results

### Patient characteristics

A total of 81 patients were included in this analysis, 46 (57%) with TV-ICDs and 35 (43%) with S‑ICDs, with a median age at implant of 16.5 and 19.0 years respectively. Median follow-up was 60.0 (45.3–60.0 months) in the TV-ICD group and 40.4 (23.5–60.0 months) months in the S‑ICD group. In the TV-ICD group 30 patients (65%) reached 5‑year follow-up; in the S‑ICD group this was the case for 12 patients (35%). Primary and secondary ICD indication did not differ significantly between the two groups, nor did the proportion of incidence of underlying pathologies described as genetic arrhythmic disease and (non)-ischaemic cardiomyopathies (Tab. 1). However, an important difference between the two groups was seen in the genetic arrhythmic patient population, as none of the patients implanted with an S‑ICD had a diagnosis of long QT syndrome, catecholinergic polymorphic ventricular tachycardia or arrhythmogenic right ventricular cardiomyopathy. Nine patients (26%) were implanted with the three-incision technique and 26 S-ICD patients (74%) with the two-incision technique. In more than half of the TV-ICD patients, 26 (56%), the pulse generator was implanted submuscularly. In 34 S-ICD patients the generator was implanted subcutaneously and in 1 patient the S‑ICD generator was placed intramuscularly under the latissimus dorsi muscle.

**Table 1:** Patient characteristics

	TV-ICD (*n* = 46)	S-ICD (*n* = 35)	*p*-value
Age at implant (years), median (IQR)	16.5 (13.0–20.2)	19.0 (16.0–23.0)	* 0.007*
Gender: Male, *n *(%)	31 (66%)	27 (75%)	0.469
Weight (kg), median (IQR)	64 (49–74)	74 (58–84)	* 0.029*
Height (cm), median (IQR)	170 (160–182)	183 (169–188)	* 0.019*
Smoking, *n* (%)	4 (9%)	3 (9%)	0.651
Atrial fibrillation, *n* (%)	2 (4%)	2 (6%)	0.583
Diabetes mellitus, *n* (%)	0 (%)	0 (%)	–
*ICD indication*			
Primary, *n *(%)	28 (61%)	19 (54%)	0.651
Secondary, *n *(%)	18 (39%)	16 (46%)	
**Diagnosis**			
*Genetic arrhythmic disease, n (%)*	40 (86%)	24 (69%)	0.051
DPP6	7 (18%)	7 (30%)	
LQTS	7 (18%)	0 (0%)	
Brugada	3 (7%)	1 (4%)	
HCM	11 (28%)	8 (33%)	
ARVC	2 (5%)	0 (0%)	
CPVT	3 (7%)	0 (0%)	
iVF	6 (17%)	8 (33%)	
*Non-ischaemic CMP, n (%)*	6 (12%)	10 (28%)	
*Congenital heart disease, n (%)*	–	1 (3%)	
*Other, n (%)*	1 (2%)	–	
**Implant technique, ** ***n*** ** (%)**			
Left generator placement	45 (98%)	35 (100%)	–
S-ICD 3‑incision	–	9 (26%)	
S-ICD 2‑incision	–	26 (74%)	
Subcutaneous implant	20 (44%)	34 (97%)	
Intramuscular implant	–	1 (3%)	
Submuscular implant	26 (56%)	–	
**Venous access in TV-ICD implant**			
Cephalic vein	29 (63%)	–	–
Subclavian vein	17 (37%)		
**ICD programming**			
Conditional zone (bpm)	180 (170–190)	200 (200–200)	*<0.05*
Unconditional zone (bpm)	222 (220–238)	250 (240–250)	* 0.05*

Results in italics indicate statistical significance

*DPP6* Dipeptidyl aminopeptidase-like protein 6 mutation, *iVF* idiopathic ventricular fibrillation*, LQTS* long QT syndrome, *HCM* hypertrophic cardiomyopathy, *ARVC* arrhythmogenic right ventricular cardiomyopathy, *CPVT* catecholinergic polymorphic ventricular tachycardia, *CMP* cardiomyopathy, *DCM* dilated cardiomyopathy

### Induced arrhythmia conversion testing

In the TV-ICD group induced arrhythmia conversion testing was performed in 42 of the 46 patients. All 42 conversion tests were successful at 15 J. Four patients were not tested due to contra-indications for conversion testing. Conversion testing was performed in 33 out of 35 S-ICD patients with a 65 J shock and was successful in standard polarity in 32 patients. One patient needed repositioning of both the S‑ICD generator and lead after a failed conversion test.

### Complications

**Fig. 2:**
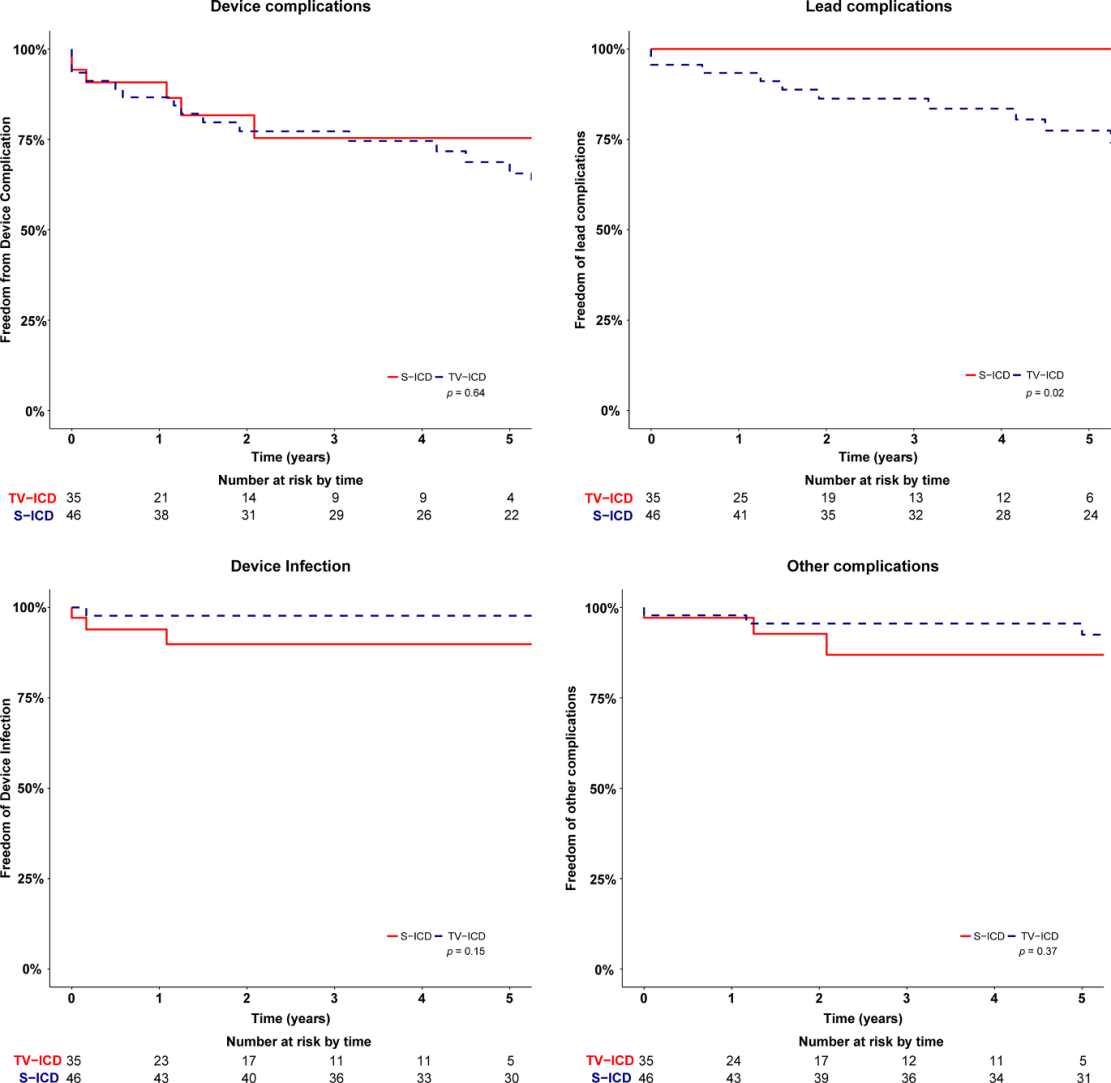
Kaplan-Meier curves for all-cause device complications, device infections and lead complications in TV-ICD and S‑ICD patients

There was no significant difference in device-related complications between TV-ICD patients and S‑ICD patients. At 5‑year follow-up, in the TV-ICD arm 13 patients experienced a device-related complication corresponding to a KM estimate of 34% (95% CI 19–49%) (Tab. 2). Six S‑ICD patients had a device-related complication; the corresponding KM estimate of the complication rate at 5‑year follow-up is 25% (95% CI 7–43%) (*p* = 0.64) (Tab. 2). The associated hazard ratio adjusted for propensity score was 1.12 (95% CI 0.41–3.06, *p* = 0.41). Analysis of different types of complications showed a significant difference in lead-related complications in favour of the S‑ICD. In the TV-ICD group, nine patients (23%) suffered a lead-related complication versus none in the S‑ICD group (*p* = 0.02) (Fig. 2). One (11%) of these nine patients was implanted with a St. Jude Medical Riata lead and two (22%) with a Medtronic Sprint Fidelis lead, both of which were on recall. Three patients in the TV-ICD group (19%) with a complication refused the required intervention and chose to have tachytherapy programmed off. One patient (6%) chose to discontinue tachytherapy because of several IAS causing severe psychological stress. Median time to lead failure in the TV-ICD group was 30 months (9–60 months).

**Table 2:** Device-related complications

*N* (%)	TV-ICD (*n* = 46)	S-ICD (*n* = 35)
Endocarditis	1 (2%)	–
Lead displacement	5 (11%)	–
Lead dysfunction	5 (11%)	–
Local device infection	–	3 (9%)
Failed conversion test	–	1 (3%)
Inadequate sensing	1 (2%)	1 (3%)
Pocket erosion	–	1 (3%)
Fistula between left mammary artery and subclavian vein	1 (2%)	–

**Fig. 3:**
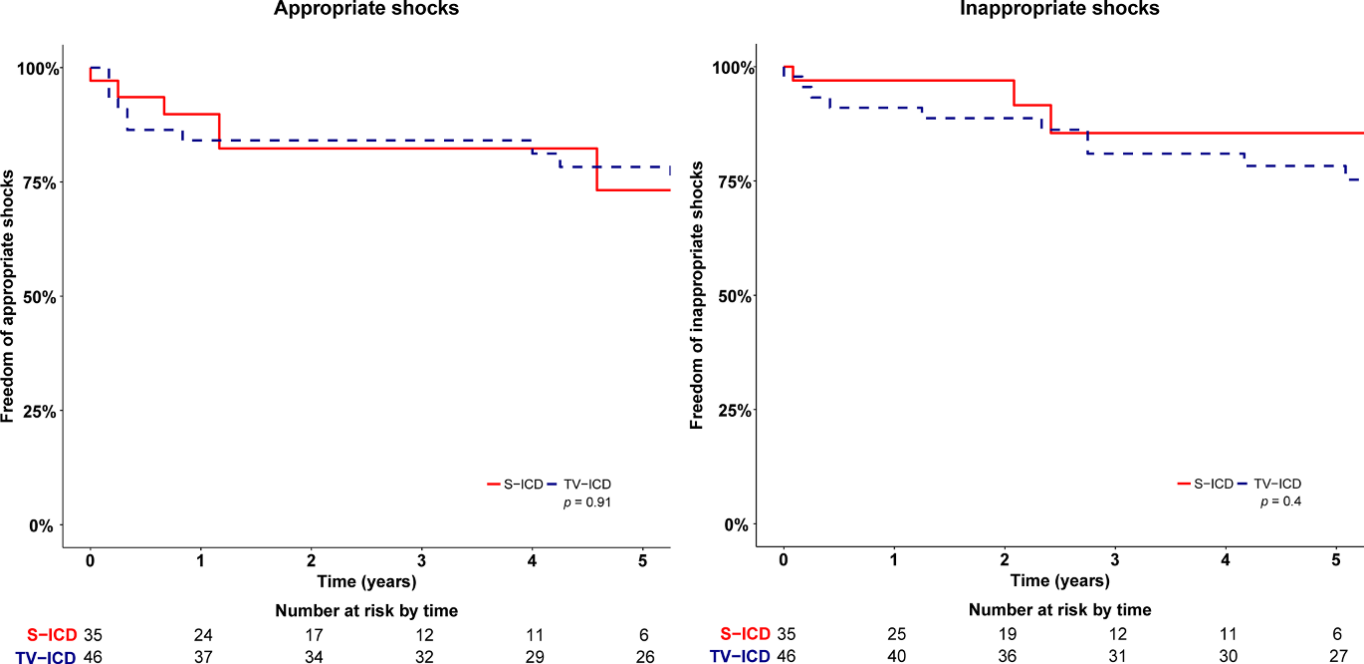
Kaplan-Meier survival curves for appropriate and inappropriate shocks in TV-ICD and S‑ICD patients

There was no significant difference in the rate of infections between TV-ICD and S‑ICD patients (*p* = 0.14). One TV-ICD patient (2%, 95% CI 0–6%) compared with three S‑ICD patients (10%, 95% CI 0–21%) underwent device extraction due to infection. The patient in the TV-ICD group was re-implanted with a TV-ICD after explantation and antibiotic treatment. All three S‑ICD patients were re-implanted with an S‑ICD after a median recovery time of 62.2 days (48.0–76.0). The period until re-implantation was bridged with a wearable cardioverter-defibrillator (Lifevest, ZOLL) in all four infection cases [11]. Other device-related complications occurred in three patients in the TV-ICD arm, KM estimate 7% (95% CI 0–8%) versus three patients in the S‑ICD group, KM estimate 13% (95% CI 0–28%) (*p* = 0.37) (Fig. 3). Details of all complications are described in Supplementary Tab. 1. Multivariable cox regression models adjusted for propensity score showed no significant difference for any subgroup of complications.

### IAS therapy

IAS occurred in 14 patients, 11 TV-ICD 19% (95% CI 7–31%) versus 3 S-ICD patients 17% (95% CI 1–33%) (*p* = 0.40). Adjusted hazard ratio for propensity score was 1.80 (95% CI 0.45–7.25, *p* = 0.41). In both groups one patient had more than one episode of IAS. Three patients in the TV-ICD group received IAS caused by lead dysfunction. All patients who experienced IAS and interventions or changes in programming are described in Supplementary Tab. 1.

### Appropriate therapy

Appropriate shocks occurred at similar rates in both groups; seven patients with a TV-ICD received one or more appropriate shocks versus five patients in the S‑ICD group (*p* = 0.92). The KM estimated rates for appropriate shocks were 25% (95% CI 11–39%) versus 27% (95% CI 6–48%) (*p* = 0.92). The adjusted hazard ratio for propensity score was 0.84 (95% CI 0.29–2.48, *p* = 0.75). First shock success rate was 83% in the S‑ICD group and 80% in the TV-ICD group, *p* = 0.76. In the TV-ICD group, three patients received successful appropriate antitachycardia pacing (ATP). The KM rate for appropriate therapy, including both ATP and shocks in TV-ICD patients, did not differ significantly, TV-ICD 27% (95% CI 13–41%) versus S‑ICD 27% (95% CI 6–48%) (*p* = 0.97). No acceleration or deterioration of ventricular arrhythmias was caused by ATP therapy in these TV-ICD patients.

### Mortality

Two patients (2.5%) died during follow-up, both in the TV-ICD group; neither death was device-related.

## Discussion

### Main findings

This study has several important findings. First, the complication rate did not differ significantly between the two devices. However, the nature of the complications did differ between the two groups. Second, lead failures requiring surgical intervention occurred only in the TV-ICD patients. However, S‑ICD patients suffered more pocket complications. Third, appropriate and inappropriate shock rates were similar in both groups.

### Complications

In the TV-ICD group the major contributor was lead complications, and in the S‑ICD group this was mainly driven by pocket complications. Lead dysfunction often caused IAS in the TV-ICD patients, resulting in such severe psychological stress that one patient refused to be protected by the ICD and had tachytherapy programmed off. Six TV-ICD patients (13%) were implanted with St. Jude Medical Riata or Medtronic Sprint Fidelis leads on which device recalls were issued. Three of these patients (50%) experienced a lead-related complication; however, two out of these three were dislocations which are not related to the reasons why the recalls were issued. Compared with the adult population incidence rates are higher, although they are driven by the same type of complications [9, 12].

The relatively high complication rate may be explained by the more slender or smaller physique and growth of the patients and higher level of daily activity resulting in more strain on the device. Additionally, in regard to this complication rate it must be noted that a learning curve is present in the S‑ICD implants [13]. Device-related complications in the S‑ICD group are expected to occur shortly after implantation, while lead-related complications in the TV-ICD group are expected to continue throughout follow-up [7, 10].

### IAS therapy

IAS therapy was similar in TV-ICD and S‑ICD patients (KM rate 22% and 14% respectively), which does not differ from IAS rates in this population described in the literature that range from 13 to 25% depending on programming, among other factors [6, 12, 14, 15]. Bordachar et al. [15] described potential benefits of the S‑ICD for this young population but warned concerning a relatively frequent occurrence of inappropriate therapy. However, this concern was not confirmed by our study. Major causes for an IAS in the TV-ICD group were lead failure and supraventricular tachycardia, in the S‑ICD group double counting of the cardiac signal (T-wave oversensing). Adequate S‑ICD screening, exercise testing before implantation and exercise templates may assist in reducing the IAS rate in this active population [16].

ATP was observed during five episodes in three TV-ICD patients but did not result in a lower appropriate shock rate compared with the S‑ICD patients. Modern-day programming would currently use more ATP programming and longer detection times, possibly reducing the appropriate shock rate in these patients.

### Clinical implications of the study

**Fig. 4:**
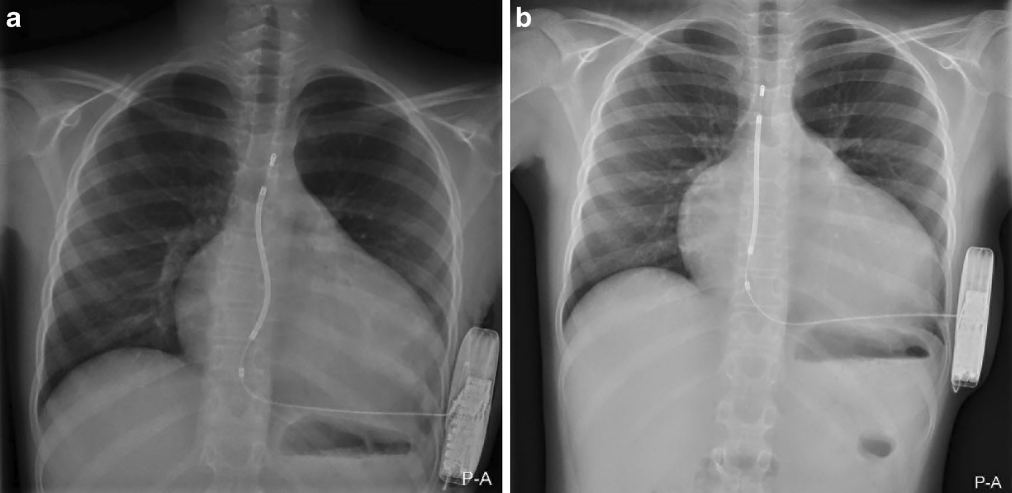
S-ICD Lead placement in a paediatric patient over time. **a** The S‑ICD lead is placed in an S‑shaped manner in a 10-year-old. **b** The same patient at the age of 12 years. The distal tip of the electrode and proximal ring of the electrode are in the same position in parts **a** and **b**, T3/4 and T10 respectively, but the S‑shape in the lead has disappeared, thus accommodating the patient’s growth

In patients where venous access needs to be preserved, is not available or when a high risk of infection is expected, the S‑ICD may provide benefits. On the other hand, the S‑ICD remains limited in its use to patients who require pacing therapy, as it does not allow pacing for bradycardia or provide ATP. Besides the underlying pathology, body growth has to be taken into consideration when implanting an ICD in paediatric and young patients. Lead placement has to allow for body growth in both TV-ICD and S‑ICD implants. This can be accommodated by implanting with loops in the lead placement, in order to prevent lead-related complications caused by mechanical stress on leads. Over time, due to the patients’ growth the S‑shape of the lead will disappear; this is demonstrated in Fig. 4. In this cohort the S‑ICD could be successfully implant in patients with a minimum weight of 30 kg. In our opinion, young patients without an indication for pacing and with a body weight above 30 kg are most suitable for S‑ICD therapy.

The infection rate is higher in the S‑ICD group, although not significantly compared with TV-ICD patients; the risk of (lead) endocarditis or the morbidity and mortality related to transvenous lead extraction is avoided. All cases of infection were non-systemic pocket infections and all S‑ICD patients in whom the S‑ICD was extracted due to infection were re-implanted with an S‑ICD therapy. This risk of infection and an additional procedure should be weighed against the advantages of a simple extraction and a low risk of systemic infection.

The sensing algorithm of the S‑ICD is able to differentiate well between supraventricular tachycardia and ventricular tachycardia. Most IAS are caused by double counting of the QRS complex or T‑wave oversensing. Novel algorithm technology is expected to further reduce the IAS rate [17].

### Limitations

This study is limited by its retrospective and observational nature and is underpowered due to the small sample size. Prospective research, with longer follow-up, in a larger and entirely homogeneous population would be valuable, but difficult to achieve in a paediatric population with low prevalence of an underlying diagnosis. A historical difference is present between the two groups, as most TV-ICD patients were implanted before the introduction of the S‑ICD. There have been considerable changes in the way a TV-ICD is programmed, as well as improvements in the design of the TV-ICD lead that have taken place over the years.

## Conclusion

In this study of paediatric and young adult patients with TV-ICD and S‑ICD the rate of device-related complications was similar, although the nature of the complications differed. No lead failures or systemic infections occurred in the S‑ICD patients. IAS rates did not differ between TV-ICD and S‑ICD patients. Management of IAS therapy differed between the two groups, as TV-ICD patients often required an intervention and S‑ICD patients with an IAS were mostly corrected by programming. Appropriate shock rates were similar in both groups, confirming the efficacy of the S‑ICD in this population. We find young patients without an indication for pacing and who have a body weight of at least 30 kg most suitable for S‑ICD therapy.

## Caption Electronic Supplementary Material

Supplementary table 1. Description of device related complications, causes of inappropriate shocks and interventions performed in S‑ICD and TV-ICD patients
