# Supplementary material for: Comparison of complications and shocks in paediatric and young transvenous and subcutaneous implantable cardioverter-defibrillator patients
Source: Neth Heart J. 2018 Oct 30;26(12):612–9. doi: 10.1007/s12471-018-1186-1 (PMC6288033; doi:10.1007/s12471-018-1186-1)
Supplement: Supplementary file 1 — Supplementary table 1. Description of device related complications, causes of inappropriate shocks and interventions performed in S‑ICD and TV-ICD patients [file 12471_2018_1186_MOESM1_ESM.doc]

**Supplementary table 1. Description of device related complications, causes of inappropriate shocks and interventions performed** in S-ICD and TV-ICD patients.

| **Patient.** | **Device related complication.** | **Implantation technique.** | **Time to complication (months)** | **Surgical intervention performed.** | **Additional surgical intervention needed.** | **Inappropriate shock therapy.** | **Time to IAS (months)** | **Number of IAS** | **Underlying mechanism of inappropriate shock.** | **Non-surgical intervention.** |
| --- | --- | --- | --- | --- | --- | --- | --- | --- | --- | --- |
| **TV-ICD 1** | No. | Left pectoral subcutaneous implant. Access left subclavian vein |  |  |  | Yes. | 28 | 1 | Sinus tachycardia after reduction of beta-blockade dosage. | Change in Fast VT zone programming 200 🡪 210 bpm. |
| **TV-ICD 2** | Endocarditis. | Right pectoral submuscular implant. Access right subclavian vein | 2 | TV-ICD extraction. | TV-ICD re-implantation after antibiotic treatment. | No. |  |  |  |  |
| **TV-ICD 3** | No. | Left pectoral subcutaneous implant. Access left cephalic vein. |  |  |  | Yes. | 33 | 4 | Sinus tachycardia during exercise. | Change in VT zone programming 180 🡪 195 bpm. |
| **TV-ICD 4** | Inadequate sensing. | Left pectoral subcutaneous implant. Access left subclavian vein. |  | None. | No. | Yes. | 2 | 2 | TWOS on SVT. | ICD programmed off on patient request. |
| **TV-ICD 5** | No. | Left pectoral subcutaneous implant. Access left subclavian vein. |  |  |  | Yes. | 5 | 6 | TWOS on SVT. | ICD programmed off on patient request. |
| **TV-ICD 6** | Fistula between left mammary artery and subclavian vein. | Left pectoral submuscular implant. Access left subclavian vein. | 37 | Stent placement in left mammary artery. | No. | Yes. | 1 | 1 | Noise, several aborted shocks. Exact cause of interference not found. No re-occurrence. | No change in programming. |
| **TV-ICD 7** | No. | Left pectoral submuscular implant. Access left subclavian vein. |  |  |  | Yes. | 2 | 2 | Sinus tachycardia during exercise |  |
| **TV-ICD 8** | Lead displacement  (riata lead) | Left pectoral submuscular implant. Access left subclavian vein. | 54 | Lead repositioning. | No. | No. |  |  |  |  |
| **TV-ICD 9** | Lead dysfunction. | Left pectoral subcutaneous implant. Access left subclavian vein. | 7 | Lead replacement. | No. | Yes. | 33 | 7 | SVT during postpartum fluxus for which 7x IAS. | No change in programming. |
| **TV-ICD 10** | Lead displacement  (fidelis lead) | Left pectoral submuscular implant. Access left subclavian vein. | 50 | Lead repositioning. | No. | Yes. | 0 | 2 | Oversensing due to lead dispositioning | Surgical intervention. |
| **TV-ICD 11** | Lead displacement | Left pectoral submuscular implant. Access left cephalic vein. | 38 | TV-ICD generator and failed lead extraction. | No. | No. |  |  |  |  |
| **TV-ICD 12** | No. | Left pectoral submuscular implant. Access left cephalic vein. |  |  |  | Yes. | 60 | 1 | ICD dysfunction, initially suspect for lead failure. | ICD pulse generator replacement. Initial leads in situ. |
| **TV-ICD 13** | Lead dysfunction – conductor fracture (fidelis lead) | Left pectoral submuscular implant. Access left subclavian vein. | 18 | No. | No. | No. |  |  |  | ICD turned off, genetic diagnoses turned out negative which was not known at the time of implant. |
| **TV-ICD 14** | Lead displacement | Left pectoral submuscular implant. Access left cephalic vein. | 5 | Lead repositioning. | No. | Yes. | 67 | 1 | Noise due to lead dysfunction. | Surgical intervention. |
| **TV-ICD 15** | Lead displacement with pericardial effusion. | Left pectoral subcutaneous implant. Access left cephalic vein. | 6 | Lead repositioning. | No. | No. |  |  |  |  |
| **TV-ICD 16** | Lead dysfunction. | Left pectoral subcutaneous implant. Access left cephalic vein. | 23 | Lead replacement. | No. | No. |  |  |  |  |
| **TV-ICD 17** | Lead fracture. | Left pectoral submuscular implant. Access left subclavian vein. | 15 | Lead replacement. | No. | Yes. | 15 | 1 | Noise due to lead fracture. | Surgical intervention. |
| **TV-ICD 18** | Lead dysfunction. | Left pectoral submuscular implant. Access left subclavian vein. |  | Lead replacement. Dysfunctioning lead abandoned. | No. | No. |  |  |  |  |
| **S-ICD 1** | No. | Subcutaneous implant 2-incision technique. |  |  |  | Yes. | 1 | 1 | TWOS during exercise (weightlifting). | Smart charge prolonged 0.91 sec. |
| **S-ICD 2** | Infection. (initial treatment conservative) | Subcutaneous implant. 2-incision technique. | 13 | S-ICD extraction. | Re-implantation of S-ICD after antibiotic treatment. | No. |  |  |  |  |
| **S-ICD 3** | Infection. (initial treatment conservative) | Subcutaneous implant. 2-incision technique. | 2 | S-ICD extraction. | Re-implantation of S-ICD after antibiotic treatment. | No. |  |  |  |  |
| **S-ICD 4** | Failed induced arrhythmia conversion test. | Subcutaneous implant. 2-incision technique. | 1 day | Repositioning of S-ICD generator intramuscular under the m. latissimus dorsi and lead repositioned and fixated using a third incision. | No. | No. |  |  |  |  |
| **S-ICD 5** | Pocket erosion. | Subcutaneous implant. 3-incision technique. |  | Pocket revision. |  | No. |  |  |  |  |
| **S-ICD 6** | Infection. | Subcutaneous implant. 3-incision technique. | 2 weeks | S-ICD extraction. | Re-implantation of S-ICD after antibiotic treatment. | No. |  |  |  |  |
| **S-ICD 7** | Inadequate sensing due to progressing CMP. | Subcutaneous implant. 2-incision technique. |  | S-ICD extraction and concomitant TV-ICD implantation. |  | Yes. | 25 | 1 | Triple counting QRS due to amplitude loss in progressing CMP. | No programmable options. Change to TV-ICD therapy. |
| **S-ICD 8** | No. | Subcutaneous implant. 3-incision technique. |  |  |  | Yes. | 29 | 2 | Oversensing ST-segment during sinus tachycardia during exercise (cycling). | S-ICD template optimized with X-ECG. Conditional zone programming changed 180 🡪 200 bpm. |

ATP = Antitachycardia pacing, IAS = Inappropriate Shock, CMP = Cardiomyopathy, S-ICD = Subcutaneous Implantable Cardioverter Defibrillator, SVT = supra ventricular tachycardia, TWOS = T-Wave Over Sensing, TV-ICD = Transvenous Implantable Cardioverter Defibrillator, VT = Ventricular Tachycardia.
